# Line × tester analysis of maize grain yield under acid and non‐acid soil conditions

**DOI:** 10.1002/csc2.20009

**Published:** 2020-02-25

**Authors:** Charles Mutimaamba, John MacRobert, Jill E. Cairns, Cosmos Magorokosho, Thokozile Ndhlela, Collis Mukungurutse, Adré Minnaar‐Ontong, Maryke Labuschagne

**Affiliations:** ^1^ Crop Breeding Institute Harare Zimbabwe; ^2^ Mukushi Seeds Harare Zimbabwe; ^3^ CIMMYT Harare Zimbabwe; ^4^ Dep. of Plant Sciences Univ. of the Free State Bloemfontein South Africa; ^5^ Chemistry and Soils Research Institute Harare Zimbabwe

## Abstract

Soil acidity has received less attention than other biophysical stresses such as drought and low N, despite accounting for a considerable reduction in maize (*Zea mays* L.) productivity in many parts of southern Africa. The line × tester mating design was used to determine the general combining ability (GCA) for grain yield of 14 maize inbred lines and the specific combining ability (SCA) of their corresponding crosses. Thirty‐three single‐cross hybrids were evaluated under acid and optimum soils across 11 environments over three seasons. Across environments, mean grain yield reduction ranged from 11 to 37% due to low pH. Additive gene action was more important than nonadditive gene action for grain yield under both soil conditions. Tester GCA effects were larger for grain yield than GCA effects of lines and SCA effects of crosses for both soil conditions. Tester GCA effects were less sensitive to environmental fluctuations than line GCA effects and SCA effects of crosses. Cross combinations with desirable SCA effects for grain yield were associated with high per se grain yield, which suggests that SCA was a good predictor of grain yield in this study. These crosses consisted of good × good and good × poor general combiners, which indicates that GCA was a good predictor of grain yield. Therefore, priority should be given for yield selection in progenies and hybridization of specific crosses with desirable SCA when breeding acid‐soil‐tolerant maize.

**Abbreviations**
CIMMYT: International Maize and Wheat Improvement Center
DR&SS: Department of Research and Specialist Services
GCA: general combining ability
SCA: specific combining ability
SS: sums of squares

## 1 INTRODUCTION

Maize (*Zea mays* L.) is grown and consumed in various forms as a major staple food and source of proteins and calories by millions of people in Southern Africa (Smale, Byerlee, & Jayne, 2011). Sustainability of maize productivity in some parts of southern Africa such as Zimbabwe is threatened by acid‐soil‐induced nutrient deficiency and heavy metal intoxication (Dhliwayo et al., 1999; Nyamangara & Mpofu, 1996). Dewi‐Hayati, Sutoyo, Syarif, and Prasetyo (2014) reported grain yield losses of 2.8–71% due to acid soils. Low soil pH can also lead to Al, Mn, or Fe toxicities (Tandzi et al., 2015; Tandzi, Mutengwa, Ngonkeu, & Gracen, 2018).

In Zimbabwe, the majority of maize is produced in rural areas by resource‐poor smallholder farmers, on granite‐derived sandveld soils which, in the higher rainfall areas, are acidic (Dhliwayo et al., 1999; Mapanda & Mavengahama, 2011; Musharo & Nyamangara, 2011; Shoko & Moyo, 2011; Tauro, Mapanda, Mtombeni, Shumba, & Dhliwayo, 2011). Acid‐soil toxicity in plants is caused by a combination of heavy metal intoxication, deficiency of essential nutrients, and acidity per se (Bian, Zhou, Sun, & Li, 2013; Tandzi, Mutengwa, Ngonkeu, & Gracen, 2018). An increase in the proportion of acidic soils leads to potential crop production problems such as low fertilizer use efficiency, Al toxicity, P unavailability, and micronutrient deficiency, and increased susceptibility to drought, which severely reduces maize yields (Bian et al., 2013). Soil acidity is less studied than other abiotic stresses such as drought and low N despite accounting for a large reduction in maize productivity in many areas of Zimbabwe.

There are various acid‐soil management strategies that can be adopted by farmers in acid‐soil environments to ameliorate soil acidity stress and enhance crop yields. These include use of improved acid‐soil‐tolerant maize varieties, P‐bearing inorganic fertilizers, agricultural lime, and other soil amendments (The et al., 2006). Soil amendments are ineffective in ameliorating subsoil acidity, though they may be temporarily effective in remedying surface soil acidity (The, Calba, Horst, & Zonkeng, 2004; The et al., 2006, 2012). Adoption and use of maize varieties bred for acid‐soil tolerance is a more viable and sustainable strategy to increase yields in acid‐soil‐prone environments (Granados, Pandey, & Ceballos, 1993; Tandzi et al., 2018; Welcker et al., 2005).

At the vegetative and reproductive stages, these biophysical stresses cause stunted seedling growth, early leaf senescence, poor flowering, and increased anthesis–silking interval or poor synchrony (Abate, Hussien, Laing, & Mengistu, 2013; Krill, Kirst, Kochian, Buckler, & Hoekenga, 2010). However, during the post‐flowering stage, continued exposure to these stresses results in reduced biomass, grain yield, and yield components such as ear prolificacy and kernel size and number (Abate et al., 2013; Krill et al., 2010). In acid tolerance breeding, putative secondary traits and yield components, combined with laboratory techniques, can be effectively used as direct selection and screening techniques (Araus, Slafer, Royo, Serret, & L. Araus, 2008). Under controlled conditions, acid soils, sand culture, solution culture, or hydroponics and in vitro techniques can be used (Deborah & Tesfaye, 2003; Dharmendra, Singh, Chauhan, & Phundan, 2011; Hede, Skovmand, & López‐Cesati, 2001; Rao, Zeigler, Vera, & Sarkarung, 1993).

Genetic variation has been detected in field trials for grain yield, plant height, days to mid‐silk, and ear prolificacy under acid and non‐acid soil conditions (Pérez Velásquez, de Souza, Narro, Pandey, & de León, 2008). Additive and dominant gene effects were more important than epistatic effects for grain yield (Pandey, Narro, Friesen, & Waddington, 2007; Pérez Velásquez et al., 2008). For plant height, additive effects were less sensitive to soil acidity than dominant gene effects. Pérez Velásquez et al. (2008) reported that additive, dominance, and epistatic effects were important in acid soil, whereas additive and dominance effects were important for prolificacy in non‐acid soils, and Pandey et al. (2007) found that the inheritance of grain yield, plant height, mid‐silk and prolificacy did not differ in acid and non‐acid soils. Narro and Arcos (2010) found that both additive and nonadditive gene action were crucial in the expression of callose content as a surrogate trait for selecting for Al tolerance. These authors recommended the use of selection and hybridization to enhance Al resistance in a maize breeding program.

Identification of the nature and magnitude of gene action governing the expression of a number of quantitative traits is achieved through combining ability analysis of a set of breeding lines. The objective of the current study was to use line × tester analysis to determine the general combining ability (GCA) of maize inbred lines and the specific combining ability (SCA) of their crosses for grain yield under acid and non‐acid soils for the purpose of identifying suitable combinations of inbred lines.

## 2 MATERIALS AND METHODS

### 2.1 Germplasm and testing environments

Three acid‐soil‐tolerant inbred lines from the International Maize and Wheat Improvement Center (CIMMYT)’s acid soil breeding program in Colombia were used as testers crossed with 11 lines from the Department of Research and Specialist Services (DR&SS) and CIMMYT–Southern Africa Regional office (SARO) to generate 33 single‐cross hybrids. Two of the lines (RS61P and NM202) were sourced from the DR&SS gene pool, whereas the rest were obtained from the CIMMYT‐Zimbabwe gene pool. The DR&SS inbred lines are susceptible to soil acidity and drought but are moderately tolerant to common maize diseases such as *Maize streak virus* (of the family Geminiviridae), gray leaf spot (*Cercospora zea‐maydis* Tehon & E. Y. Daniels), Turcicum leaf blight [*Exserohilum turcicum* (Pass.) K. J. Leonard & Suggs], and leaf rust (*Puccinia sorghi* Schwein.). The CIMMYT‐Zimbabwe inbred lines, though susceptible to soil acidity, are tolerant to drought and common maize diseases. The testers are tolerant to drought and soil acidity, especially Al toxicity, but are nevertheless susceptible to common maize diseases. The 33 hybrids, together with two commercial checks (SC633 and SC727) were evaluated over three years (2011–2012, 2012–2013, and 2013–2014) at four locations in Zimbabwe: Bindura, Domboshava, Makoholi, and Marondera (Table 1). The commercial checks were hybrids adapted to and produced in the region where the trials were planted, but they were not classified as acid soil tolerant. They were used for comparison with the crosses. Rainfall varied from 339 mm at Makoholi to 1,100 mm at Marondera in the 2011–2012 season, 417 mm at Makoholi to 820 mm at Marondera in 2012–2013, and 693 mm at Domboshava to 1,040 mm at Marondera in 2013–2014 (Table 1). Soil pH differences between limed and unlimed blocks varied from 0.6 to 1.2 points, where pH level ranged from 3.7 (extremely acidic) at Marondera to 5.6 at Bindura (Table 2). Available P ranged from 11 mg kg^−1^ at Domboshava to 65 mg kg^−1^ at Marondera. Soil nutrient levels were generally higher in heavy‐textured soils at Bindura than light sandy soils at Domboshava, Marondera, and Makoholi.

**TABLE 1:** Description of testing environments and rainfall amounts received over three seasons (October–May)

				Total rainfall		
Location	Latitude	Longitude	Altitude	2011–2012	2012–2013	2013–2014
			m asl	mm		
Bindura	17°12′ S	31°10′ E	1,136	781.0	781.0	1,073.5
Harare	17°43′ S	31°05′ E	1,500	533.5	747.0	955.7
Domboshava	17°36′ S	31°08′ E	1,538	846.6	709.0	693.0
Makoholi	19°50′ S	30°46′ E	1,189	339.2	417.0	838.0
Marondera	18°10′ S	31°29′ E	1,617	1,098.9	821.7	1,039.5

**TABLE 2:** Chemical and physical characteristics of the top 30 cm of the soil profile at each site

		Parameter^a^						
Site	Treatment	pH (CaCl_2_)	P_2_O_5_	Ca	K	Mg	Color	Texture
			mg kg^−1^	cmol_c_ kg^−1^				
2011–2012								
Marondera	Low pH	3.7	48	0.7	0.1	0.3	PB	MgS
Domboshava	Low pH	4.6	22	0.7	0.1	0.3	PB	MgS
2012–2013								
Marondera	Low pH	4.2	48	0.6	0.1	0.3	PB	MgS
	Optimal pH	5.1	45	1.0	0.1	0.5	PB	MgS
Domboshava	Low pH	4.2	11	0.4	0.1	0.3	PB	MgS
	Optimal pH	5.4	17	0.7	0.1	0.6	PB	MgS
Bindura	Low pH	4.5	29	14.1	0.7	4.7	DB	MgSCL
	Optimal pH	4.3	33	16.1	0.4	5.1	DB	MgSCL
Makoholi	Low pH	5.1	26	0.4	0.1	0.3	PB	MgS
	Optimal pH	5.1	27	0.4	0.1	0.3	PB	MgS
2013–2014								
Bindura	Low pH	4.5	42	12.0	0.5	3.6	DB	MgSCL
	Optimal pH	5.6	36	13.2	0.5	3.6	DB	MgSCL
Makoholi	Low pH	4.5	33	0.7	0.1	0.3	PB	MgS
	Optimal pH	5.1	19	0.6	0.1	0.3	PB	MgS

^a^P_2_O_2_, available phosphate (resin method); pH, alkalinity/acidity; na, not analyzed; PB, pale brown; DB, dark brown; MgS, medium‐grained sand; MgLS, medium‐grained loamy sand; MgSCL, medium‐grained sandy clay loam. Soil pH: <4.0 = extremely acidic; 4.0–4.5 = very strongly acidic; 4.5–5.0 = strongly acidic; 5.0–5.5 = medium acidic; 5.5–6.0 = slightly acidic.

### 2.2 Experimental design and trial management

The experimental field was subdivided into two equal blocks in each environment to facilitate separate evaluation of two sets of genotypes, one under naturally acidic (low pH) or unlimed soil conditions, and the other one under optimal or limed conditions. The 33 crosses and two commercial checks were planted in a five‐by‐seven α‐lattice design with three replications at each environment with two treatments: low pH and optimal soil conditions. Checks were randomized as part of the lattice design. Each plot consisted of a 4‐m single row, with 75‐cm inter‐row spacing and 25‐cm in‐row spacing. Two seeds were hand planted per station and thinned to 53,000 plants ha^−1^ at 3 wk after crop emergence. Trials were planted with border rows to minimize border effects.

A minimum of five subsamples of soil were taken per site prior to planting and liming based on soil uniformity. Environment‐specific fertilizer recommendations and manual and/or chemical control of weeds and pests were used for each trial. A total of 400 kg ha^−1^ of Compound D (equivalent to 28 kg N ha^−1^, 56 kg P_2_O_5_ ha^−1^, and 28 kg K_2_0 ha^−1^) was applied as basal dressing at Bindura, Domboshava, and Marondera, whereas 300 kg ha^−1^ Compound D (equivalent to 21 kg N ha^−1^, 42 kg P_2_O_5_ ha^−1^, and 21 kg K_2_O ha^−1^) was applied as basal dressing at Makoholi. Ammonium nitrate (34.5% N) was split applied as top‐dressing at 400 kg ha^−1^ at Bindura, Domboshava, and Marondera, and 300 kg ha^−1^ at Makoholi. Prior to planting, agricultural lime was applied based on soil test results at the optimum environments.

### 2.3 Data collection

Grain yield per hectare for each plot was estimated from the field weight by adjusting the grain moisture to 12.5%. At harvest, edge plants from both sides of rows were removed from all trials to avoid border effects.

### 2.4 Statistical analysis

Analysis of variance was done and combining ability effects determined using AGROBASE version II (Agronomix Software, 2010), whereas combining ability variances were determined using Analysis of Genetic Designs with R for Windows (AGD‐R) (Rodríguez, Alvarado, Pacheco, Burgueño, & Crossa, 2015). The GCA effects of lines and testers and SCA effects of hybrids were estimated using line × tester analysis (Dabholkar, 1999; Kempthorne, 1957), where females were designated as lines and males as testers. Genotypes (lines, testers, and hybrids) and environments were treated as fixed, whereas interactions (site × line, site × tester, line × tester, and site × line × tester), replication, and block were treated as random effects. The underlying statistical model for line × tester analysis was assumed in the combining ability analysis (Arunachalam, 1974; Dabholkar, 1999):

$$\;Y_{\mathrm{ijk}}=\;\mu +g_{i}+g_{j}+s_{\mathrm{ij}}+r_{k}+e_{\mathrm{ijk}}$$

where *Y_ijk_* is the mean value of a character measured on cross *i* × *j* in the *k*th replication; *g_i_* is the GCA effect of the *i*th parent; *g_j_* is the GCA effect of the parent *j*; *s_ij_* is the SCA effect of cross *i* × *j*; *r_k_* is the replication effect; *e_ijk_* is the environmental effect peculiar to the (*ijk*)th individual; and μ is the population mean effect.

To estimate the GCA effect of the *i*th line,

$${\hat{g}}_{i}=\frac{Y_{i\ldots }}{\mathrm{rf}}-\frac{Y_{\ldots }}{\mathrm{rmf}}$$

To estimate the GCA effect of the *j*th tester,

$${\hat{g}}_{j}=\frac{\;Y_{.j.}}{\mathrm{rm}}-\frac{Y_{\ldots }}{\mathrm{rmf}}$$

To estimate of SCA effect of the *i* × *j* cross,

$${\hat{S}}_{\mathrm{ij}\;}\frac{\;Y_{\mathrm{ij}.}}{r}-\frac{Y_{i\ldots }}{\mathrm{rf}}-\frac{Y_{.j.}}{\mathrm{rm}}+\frac{Y_{\ldots }}{\mathrm{rmf}}$$

where *r* is the number of replications; *m* is the number of male parents (testers); and *f* is the number of female parents (lines). The lines and testers represented both heterotic groups A and B (data not shown), but all lines were crossed to all testers irrespective of heterotic groups, to follow a line × tester design.

To determine significant differences within treatments and between treatments, ANOVA was done for each treatment separately, and then as a combined ANOVA per environment for two treatments (optimal and acid soil). This was followed by combined ANOVA for optimal and acid soil treatments for Bindura 1 and 2 combined, Marondera and Dombashava combined, and Bindura 2 and Makoholi combined.

## 3 RESULTS

### 3.1 Mean yield performance of hybrids and broad sense heritability of grain yield under acid and optimal soil conditions

The effect of genotype on yield was significant for all trials (data not shown). Low soil pH significantly reduced grain yield relative to the control in all environments, with the exception of Bindura 1 (Table 3). Grain yield was reduced by 11% in Marondera, 37% in Domboshava, and 17% in Makoholi 2 and Bindura 2. Mean grain yield under low pH ranged from 0.4 to 3.6 t ha^−1^ with an average of 1.6 t ha^−1^ at Bindura 2, to 2.4 to 6.9 t ha^−1^ with an average of 5.2 t ha^−1^ at Bindura 1 (Table 3). Under optimum soils, mean grain yield varied from 0.3 to 3.1 t ha^−1^ with an average of 1.8 t ha^−1^ at Marondera to 2.6 to 13.4 t ha^−1^ with an average of 7.1 t ha^−1^ at Domboshava. The highest yielding experimental hybrids were C12 × A1 at Marondera and C8 × A2 at Domboshava under low pH, and C5 × A1 at Marondera and C6 × A1 at Domboshava under optimum conditions (Table 3). The top‐yielding experimental hybrids were C12 × A3 at Makoholi 1 and C6 × A1 at Bindura 1 under low pH, whereas under optimum conditions, it was C8 × A1 at Bindura 1. The highest yielding experimental hybrids were C5 × A1 at Makoholi 2 and C6 × A1 at Bindura 2 under low pH, and C14 × A1 under optimum soils at Makoholi 2 and Bindura 2. The commercial check variety SC727 outyielded all hybrids at Domboshava and Bindura 2 under low pH and at Domboshava and Bindura 1 and 2 under optimum conditions (Table 3). Broad‐sense heritability was low (<.40) at Marondera and Bindura 2 for both treatments (Table 3). It was. however, moderate to high (between .40 and .70) for both treatments at Domboshava, Makoholi 2 and Bindura 2, and under low pH at Makoholi 1.

**TABLE 3:** Mean grain yield and broad‐sense heritability of maize hybrids for different locations and seasons under acid and optimal soil conditions

	Marondera	Domboshava		Makoholi 1		Bindura 1		Makoholi 2		Bindura 2	
Hybrid	LpH^a^	Op^b^	LpH	Op	LpH	LpH	Op	LpH	Op	LpH	Op
	t ha^−1^										
C4 × A1	2.2	1.5	2.7	6.9	1.6	5.7	3.2	1.7	2.6	3.3	1.7
C4 × A2	1.6	2.6	3.4	6.1	1.6	6.0	3.9	1.7	1.7	1.2	1.7
C4 × A3	2.0	2.4	2.8	6.3	1.7	5.7	3.2	1.3	1.3	1.1	1.2
C5 × A1	2.0	3.1	3.7	4.8	1.5	6.0	5.6	2.7	3.6	1.5	2.9
C5 × A2	1.6	1.1	3.5	5.9	0.8	6.2	5.6	2.2	2.4	1.7	1.3
C5 × A3	1.0	1.5	4.2	4.8	1.9	3.0	3.7	2.2	1.2	2.2	1.4
C6 × A1	2.2	1.8	5.8	10.1	1.4	6.9	4.8	1.7	2.8	3.4	4.3
C6 × A2	2.2	2.5	5.1	7.6	1.9	6.6	3.7	2.6	2.6	2.4	2.8
C6 × A3	1.1	0.3	3.1	7.8	1.6	6.5	5.2	1.7	1.8	1.6	2.6
C7 × A1	1.9	1.9	4.3	8.4	1.9	6.3	5.4	1.7	2.5	3.0	3.8
C7 × A2	1.3	1.2	5.2	7.3	1.7	4.7	5.2	1.1	1.2	1.1	1.3
C7 × A3	1.5	1.9	4.0	6.1	2.0	5.8	5.5	2.0	3.0	1.0	1.8
C8 × A1	2.1	2.8	5.7	7.8	1.5	5.4	6.1	1.8	1.8	1.9	2.2
C8 × A2	1.7	1.9	6.6	8.0	1.5	5.0	5.7	1.8	1.7	0.9	1.2
C8 × A3	0.9	1.7	4.7	5.6	1.5	4.2	4.1	1.9	2.7	1.5	2.3
C9 × A1	1.5	1.6	4.6	9.5	1.9	5.5	5.4	2.2	2.8	1.7	2.6
C9 × A2	1.4	1.8	4.5	7.3	1.6	5.0	4.8	1.5	3.0	1.8	3.3
C9 × A3	0.6	1.4	5.2	9.6	1.5	4.1	3.9	2.0	2.5	2.8	2.3
C10 × A1	2.4	2.4	4.2	9.8	1.8	2.4	6.0	2.5	1.5	2.7	3.3
C10 × A2	0.9	1.1	3.8	6.8	1.2	4.8	4.1	1.7	1.9	2.8	1.8
C10 × A3	1.5	1.2	1.9	2.6	1.9	3.5	3.9	0.5	0.8	0.4	2.4
C11 × A1	1.5	2.0	5.2	7.7	1.8	6.3	4.2	2.6	2.9	1.6	1.6
C11 × A2	2.0	1.1	5.6	6.0	1.6	5.4	4.2	2.1	2.2	1.4	2.1
C11 × A3	1.4	1.2	5.7	5.3	1.5	4.7	6.9	1.8	3.3	1.3	2.8
C12 × A1	2.4	2.5	5.9	8.0	1.9	3.6	5.0	2.0	2.3	2.9	2.8
C12 × A2	1.1	1.6	5.6	5.7	1.8	5.7	5.8	2.0	2.5	2.3	2.0
C12 × A3	1.0	2.4	3.5	7.0	2.2	5.9	5.8	1.5	2.2	2.0	1.9
C13 × A1	1.7	1.7	4.4	5.5	2.1	5.7	5.6	1.8	2.1	2.6	3.3
C13 × A2	1.2	1.9	3.9	4.4	1.3	6.2	4.6	1.6	2.6	2.4	2.0
C13 × A3	1.5	1.6	3.0	4.2	2.1	5.4	4.9	1.1	1.2	1.2	3.1
C14 × A1	2.1	2.8	5.8	9.5	1.4	4.7	4.7	2.0	3.7	2.7	5.4
C14 × A2	1.5	2.3	5.7	8.0	1.9	6.2	5.3	2.7	2.6	2.9	1.6
C14 × A3	1.6	2.0	4.5	6.9	2.0	3.9	4.8	2.2	2.2	1.3	1.5
SC633	1.6	0.8	3.3	7.3	1.7	5.1	4.0	2.1	2.0	3.0	2.0
SC727	1.9	2.4	7.2	13.4	1.9	3.9	7.6	1.8	3.4	3.6	5.4
Mean	1.6	1.8	4.5	7.1	1.7	5.2	4.9	1.9	2.3	2.0	2.4
Heritability (broad sense)	.30	.34	.49	.62	.42	.38	.30	.52	.50	.53	.49
LSD (0.05) (hybrid)	0.9	1.2	1.6	1.7	0.5	1.8	1.9	0.6	1.0	1.0	1.4
LSD (0.05) (treatment)	0.1	0.3	0.0	0.4	0.2	0.3					

^a^LpH, low pH (acid soils).

^b^Op, optimum soils.

Grain yield was significantly reduced by low pH relative to the control across seasons at Bindura, and across environments at Marondera and Domboshava, and Bindura and Makoholi (Table 4). Low pH reduced grain yield by 13% across seasons at Bindura 1 and 2, by 31% across environments at Marondera and Domboshava, and by 17% across environments at Bindura 2 and Makoholi 2. Mean grain yield under low pH varied from 2.1 to 4.3 t ha^−1^ with an average of 3.4 t ha^−1^ across seasons at Bindura, 1.8 to 4.6 t ha^−1^ with an average yield of 3.1 t ha^−1^ across environments at Marondera and Domboshava, and 0.4 to 2.8 t ha^−1^ with an average of 2.0 t ha^−1^ across environments at Bindura and Makoholi (Table 4). Under optimum soils, mean grain yield averaged 3.9 t ha^−1^ with a range of 2.2 to 6.5 t ha^−1^ across seasons at Bindura, 4.5 t ha^−1^ with a range of 1.9 to 7.9 t ha^−1^ across environments at Marondera and Domboshava, and 2.4 t ha^−1^ with a range of 1.2 to 4.6 t ha^−1^ across environments at Bindura and Makoholi. The highest yielding experimental hybrids under low pH were C10 × A1 across seasons at Bindura, C11 × A1 across environments at Marondera and Domboshava, and C14 × A2 across environments at Bindura and Makoholi (Table 4). Under optimum soils, the top‐yielding experimental hybrids were C6 × A1 across seasons at Bindura and C14 × A1 across environments at Marondera and Domboshava, and Bindura and Makoholi. SC727 was the highest yielding hybrid under low pH across environments at Marondera and Domboshava and under optimum soils across seasons at Bindura and across environments at Marondera and Domboshava (Table 4).

**TABLE 4:** Mean grain yield (t ha^−1^) of maize hybrids combined across locations and seasons under acid and optimal soil conditions

	Across seasons			Across sites		
	Bindura 1 and 2		Marondera and Domboshava		Bindura 2 and Makoholi	
Hybrid	Low pH	Optimal	Low pH	Optimal	Low pH	Optimal
	t ha^−1^					
C4 × A1	3.3	3.8	3.1	5.1	2.5	2.2
C4 × A2	2.5	3.9	3.2	4.3	1.4	1.7
C4 × A3	2.2	3.5	2.8	4.0	1.3	1.3
C5 × A1	3.5	4.5	3.9	5.3	2.1	3.3
C5 × A2	3.7	3.7	4.1	5.0	2.0	1.9
C5 × A3	3.0	2.2	2.8	3.7	2.2	1.3
C6 × A1	4.1	5.6	3.0	6.0	2.5	3.5
C6 × A2	3.1	4.7	3.0	4.6	2.5	2.7
C6 × A3	3.4	4.5	2.9	5.5	1.7	2.2
C7 × A1	4.2	5.1	3.3	6.1	2.3	3.1
C7 × A2	3.2	3.0	2.3	4.0	1.1	1.2
C7 × A3	3.3	3.8	1.8	1.9	1.5	2.4
C8 × A1	4.0	3.8	2.0	4.2	1.8	2.0
C8 × A2	3.3	3.1	2.5	4.5	1.4	1.4
C8 × A3	2.8	3.3	2.4	4.3	1.7	2.5
C9 × A1	3.5	4.1	3.4	4.9	1.9	2.6
C9 × A2	3.3	4.2	3.8	3.6	1.7	3.2
C9 × A3	3.4	3.2	3.5	3.2	2.4	2.4
C10 × A1	4.3	2.8	2.9	4.0	2.6	2.4
C10 × A2	3.5	3.3	3.0	3.5	2.3	1.9
C10 × A3	2.1	2.9	2.6	3.2	0.4	1.6
C11 × A1	2.9	3.9	4.2	5.3	2.1	2.3
C11 × A2	2.8	3.8	3.4	3.7	1.8	2.2
C11 × A3	4.1	3.7	2.2	4.7	1.6	3.0
C12 × A1	3.9	3.2	4.0	5.9	2.4	2.6
C12 × A2	4.1	3.8	3.6	5.1	2.1	2.2
C12 × A3	3.9	3.9	2.1	4.1	1.8	2.1
C13 × A1	4.1	4.5	3.1	3.6	2.2	2.7
C13 × A2	3.5	4.1	2.6	3.1	2.0	2.3
C13 × A3	3.0	4.2	2.2	2.9	1.1	2.1
C14 × A1	3.7	5.1	3.9	6.2	2.4	4.6
C14 × A2	4.1	3.9	3.6	5.2	2.8	2.1
C14 × A3	3.1	2.7	3.1	4.4	1.7	1.9
SC633	4.1	3.0	2.4	4.1	2.6	2.0
SC727	3.8	6.5	4.6	7.9	2.7	4.4
Mean	3.4	3.9	3.1	4.5	2.0	2.4
LSD (0.05)^a^	1.00 (0.23)	0.91 (0.21)	0.72 (0.16)			
Genotype	**	**	***	***	***	***
G × E^b^	NS^†^	NS	***	***	**	**

^a^Between treatment LSD is in parenthesis.

^b^G × E, genotype × environment interaction.

^**^, ^***^ Significant at the .01 and .001 probability levels, respectively. ^†^NS, not significant.

At Marondera under low‐pH conditions, 13 of the crosses yielded higher than check SC633, and 10 crosses higher than check SC727, but none of the differences were significant (Table 3). Under optimal conditions, eight crosses yielded higher than check SC727, but the differences were not significant. At Domboshava, under low pH, all hybrids had lower yield than check SC727, but for nine crosses, the difference was not significant. Thirteen crosses yielded significantly higher than check SC633. Under optimum conditions at Domboshava, check SC727 outyielded all the crosses significantly. Five of the crosses significantly outyielded check SC633. At Makoholi 1 under low pH, five crosses yielded higher than check SC727 and 15 crosses yielded higher than check SC633, but none of the differences were significant. At Bindura 1 (low pH), only four crosses yielded less than check SC727, and 20 crosses yielded higher than check SC633, but none of the differences were significant. At Bindura under optimal conditions, only five crosses had yield not significantly lower than check SC727, and three crosses yielded significantly higher than check SC633. At Makoholi 2 under low pH, nine crosses yielded higher than check SC633, and 16 crosses higher than check SC727, but none of the differences were significant. Under optimum conditions, five of the crosses had significantly higher yield than check SC633, but none had significantly higher yield than check SC727. At Bindura 2 under low pH conditions, no cross had higher yield than check SC727, and three crosses had higher yield than check SC633 (not significant). Under optimum conditions, three crosses yielded significantly higher than check SC633, but SC727 had the highest yield of all the entries.

In the combined trials (Table 4), check SC727 generally ranked first for yield in all trials, indicating that it was the best, without selection for low pH tolerance. In the case of check SC633, many of the crosses yielded better than this check.

### 3.2 Estimates of GCA and SCA variance for grain yield under acid and optimal soil conditions

The effect of environment, genotype, and line and tester GCA variance were highly significant (*P* ≤ .001) for grain yield and significant (*P* ≤ .01) for replications and SCA (Table 5). The effects of environment × genotype, environment × line GCA, and environment × tester GCA interaction were highly significant (*P* ≤ .001), and that of environment × SCA significant (*P* ≤ .05) for grain yield. The GCA variance for yield of lines and testers were 3.6 and 21.7 times larger than SCA variance, respectively, whereas tester GCA variance was six times larger than line GCA variance (Table 5). The environment × GCA variance of lines was about four times larger than the environment × GCA variance of testers. Environment sums of squares (SS) were eight times larger than genotype SS, whereas SS for tester GCA slightly exceeded SS for line GCA variance (Table 5). The SS for environment, genoytpye, and genotype × environment interaction were 41.7, 5.1, and 16.2% of total SS. The SS for tester GCA were more than twice that of SCA variance, whereas SS for line GCA were almost twice that of SCA. The SS for environment × SCA were four times higher than the SS for environment × GCA of testers. The SS for environment × GCA of lines were almost twice that of SS for environment × GCA of testers.

**TABLE 5:** Analysis of variance for general combining ability (GCA) effects of lines and testers and specific combining ability (SCA) effects of crosses for grain yield across acid and optimal soil conditions over all environments

Source of variation	df	SS^a^	MS^b^
Environments (Env)	10	1,605.87	160.59***
Replications	22	50.13	2.28**
Genotypes (Gen)	32	197.38	6.17***
GCA_line_	10	70.84	7.08***
GCA_tester_	2	86.47	43.24***
SCA	20	39.87	1.99^*^
Env × Gen	320	624.51	1.95***
Env × GCA_line_	100	273.71	2.74***
Env × GCA_tester_	20	70.26	3.51***
Env × SCA	200	280.54	1.40*
Residuals	506	551.83	1.09
Total	1,242	3,851.42	

^a^SS, sums of squares.

^b^MS, mean squares.

^*^, ^**^, ^***^ Significant at the .05, .01, and .001 probability levels, respectively.

### 3.3 Estimates of GCA effects for grain yield under acid and optimal soil conditions

The highest positive GCA effect under low pH was obtained from line C8, whereas A1 had the highest positive GCA effect among testers (Table 6). Under optimum soil conditions, C9 and A1 had the highest positive GCA effects among lines and testers, respectively. Among lines, C14 contributed 20% of all positive GCA effects under both low‐pH and optimum soils. It was closely followed by line C6, which contributed 17% of all positive GCA effects under both environments. Under low‐pH and optimum soils, A1 contributed 69% of all positive GCA effects, whereas A3 provided 60% of all negative GCA effects among testers. In addition, 44% of total positive GCA effects for A1 were observed under low pH. However, ∼33% of significant GCA effects for A2 were positive and observed under low pH. About 80% of total positive GCA effects for line C12, and 50% of respective total positive GCA effects for lines C6 and C14, were observed under low pH. Under both soil conditions, line C10 was associated with a combination of the highest number of negative GCA effects and a lack of positive GCA effects, whereas tester A3 contributed 60% of all negative GCA effects.

**TABLE 6:** Estimates of line and tester general combining ability (GCA) effects for maize grain yield under acid and optimal soil conditions

	Marondera		Domboshava		Makoholi 1	Bindura 1		Makoholi 2			Bindura 2	
Genotype	Low pH	Optimal	Low pH	Optimal	Low pH	Low pH	Optimal	Low pH		Optimal	Low pH	Optimal
Line												
C4	0.34**	0.32*	−1.49**	−0.47	−0.09**	−1.45**	0.57*	−0.30**	−0.38**		−0.07	−0.82**
C5	−0.09	0.04	−0.67**	−1.73**	−0.30**	0.10	−0.18	0.49**	0.15*		−0.16	−0.50**
C6	0.24**	−0.33**	0.17	1.59**	−0.07**	−0.32	1.40**	0.11**	0.10		0.52**	0.84**
C7	−0.02	−0.23	0.01	0.39	0.16**	0.53*	0.38	−0.27**	−0.08		−0.23**	−0.06
C8	−0.01	0.26*	1.17**	0.26	−0.23**	0.43	−0.37	−0.04	−0.20**		−0.53**	−0.46**
C9	−0.42**	0.05	0.29	1.92**	−0.02	−0.17	−0.37	0.02	0.46**		0.12	0.36*
C10	0.03	−0.31*	−1.17**	−0.50	−0.08**	−0.23	−1.67**	−0.29**	−0.86**		0.02	0.10
C11	0.04	−0.44**	1.01**	−0.55**	−0.09**	0.23	0.20	0.30**	0.53**		−0.52**	−0.21
C12	−0.09	0.29*	0.51*	0.02	0.27**	0.67*	−0.19	−0.05	0.05		0.41**	−0.15
C13	−0.15*	−0.15	−0.69**	−2.20**	0.43**	0.15	0.53	−0.39**	−0.31**		0.09	0.43*
C14	0.12	0.49**	0.86**	1.28**	0.04	0.05	−0.32	0.42**	0.55**		0.34**	0.47**
LSD (0.05)	0.75	1.00	1.31	1.43	0.40	1.45	1.53	0.50	0.78		0.84	1.10
Tester												
A1	0.40**	0.41**	0.28**	1.12**	0.00	0.08	0.21**	0.18**	0.31**		0.51**	0.70**
A2	−0.10**	−0.13**	0.33**	−0.24**	−0.09**	0.38**	−0.06	0.03**	−0.05**		−0.05	−0.45**
A3	−0.30**	−0.27**	−0.60**	−0.88**	0.08*	−0.46**	−0.15	−0.21**	−0.26**		−0.47**	−0.24**
LSD (0.05)	0.39	0.52	0.68	0.74	0.212	0.76	0.79	0.26	0.40		0.43	0.57

^*^, ^**^ Significant at the .05 and .01 probability levels, respectively.

### 3.4 Estimates of SCA effects for grain yield under acid and optimal soil conditions

Significant positive SCA effects were detected for the crosses C11 × A2 under low pH, C5 × A1 and C6 × A2 under optimum soils at Marondera, and C9 × A3 and C10 × A1 at Domboshava under low pH (Table 7). The SCA effects were significant and positive under low pH for C5 × A3, C6 × A2, C9 × A1, C10 × A1, C10 × A3, C11 × A1, C12 × A3, C13 × A2, and C14 × A2 at Makoholi 1 and C11 × A3 at Bindura 1. Significant positive SCA effects were observed under low pH for C6 × A2, C7 × A3, C8 × A3, C7 × A1, C9 × A3, C10 × A1, and C14 × A2 at Makoholi 2, and C4 × A1, C5 × A3, C7 × A1, C9 × A3, C10 × A1, C11 × A2, and C14 × A2 at Bindura 2. The SCA effects were significant and positive under optimum soils for C5 × A1, C7 × A3, C8 × A3, C10 × A2, C11 × A3, C13 × A2, and C14 × A1 at Makoholi 2 and C9 × A2 and C14 × A1 at Bindura 2. Significant negative SCA effects were detected for C11 × A1 and C6 × A3 under optimum soils at Marondera, and C5 × A1 and C10 × A3 under low pH at Domboshava. The SCA effects were significant and negative for C5 × A2, C6 × A1, C9 × A3, C10 × A2, C11 × A3, C13 × A3, and C14 × A1 under low pH at Makoholi 1, and C5 × A3 under low pH at Bindura 1. Significant and negative SCA effects were observed for C6 × A1, C7 × A2, C8 × A1, C9 × A2, C10 × A3, and C14 × A1 at Makoholi 1, C4 × A2, C5 × A1, C7 × A2, C9 × A1, and C10 × A3 at Bindura 2 under low pH. The SCA effects were significant and negative under optimum soils for C5 × A3, C7 × A2, C8 × A1, C11 × A2, and C13 × A3 at Makoholi 2, and C11 × A1 at Bindura 2.

**TABLE 7:** Estimates of specific combining ability (SCA) effects for grain yield under acid and optimal soil conditions across seasons

	Marondera		Dombishava		Makoholi1	Bindura 1		Makoholi 2		Bindura 2	
Cross	LpH^a^	Op^b^	LpH	Op	LpH	LpH	Op	LpH	Op	LpH	Op
C4 × A1	−0.11	−1.06**	−0.64	−0.57	0.02	−0.15	−0.44	−0.04	0.40	0.92**	−0.59
C4 × A2	−0.26	0.57	−0.08	0.12	0.03	−0.17	0.54	0.06	−0.11	−0.66*	0.66
C4 × A3	0.37	0.49	0.72	0.45	−0.05	0.31	−0.10	−0.02	−0.29	−0.26	−0.07
C5 × A1	0.07	0.81*	−1.45*	−0.37	0.10	0.90	0.42	0.14	0.89**	−0.87**	0.32
C5 × A2	0.16	−0.71	0.96	−0.62	−0.49**	0.73	0.69	−0.22	0.05	−0.01	−0.14
C5 × A3	−0.23	−0.10	0.49	0.99	0.39**	−1.64*	−1.10	0.09	−0.95**	0.87**	−0.18
C6 × A1	−0.01	−0.17	0.49	0.88	−0.28**	0.15	0.04	−0.49*	0.07	0.41	0.34
C6 × A2	0.42	1.11**	−0.68	0.09	0.39**	−0.44	−0.79	0.55**	0.25	−0.04	0.00
C6 × A3	−0.40	−0.94*	0.18	−0.97	−0.12	0.29	0.75	−0.06	−0.33	−0.37	−0.35
C7 × A1	−0.08	−0.21	0.01	−0.49	0.06	0.63	−0.19	−0.10	−0.05	0.76**	0.75
C7 × A2	−0.20	−0.35	0.30	0.37	−0.12	−1.27	−0.10	−0.49**	−0.98**	−0.54*	−0.52
C7 × A3	0.28	0.55	−0.31	0.12	0.05	0.63	0.29	0.60**	1.02**	−0.21	−0.23
C8 × A1	0.11	0.24	−0.44	−0.26	0.00	0.42	0.59	−0.21*	−0.57*	−0.07	−0.36
C8 × A2	0.22	−0.07	1.08	0.58	0.07	−0.26	0.42	−0.09	−0.33	−0.45	−0.27
C8 × A3	−0.33	−0.17	−0.64	−0.32	−0.07	−0.16	−1.01	0.29**	0.90*	0.52	0.63
C9 × A1	−0.08	0.24	−0.45	−0.48	0.22*	0.54	0.48	0.10	−0.37	−0.93**	−0.84
C9 × A2	0.36	−0.01	−1.25	−0.57	0.03	−0.26	0.13	−0.41**	0.35	−0.24	1.06^*^
C9 × A3	−0.28	−0.23	1.70**	1.04	−0.25**	−0.28	−0.61	0.31**	0.02	1.17**	−0.22
C10 × A1	0.39	0.40	2.28**	0.63	0.17**	−1.26	1.10	0.74**	−0.20	0.23	0.09
C10 × A2	−0.61	−0.28	0.66	0.13	−0.31**	0.85	−0.48	0.11	0.58*	0.92**	−0.24
C10 × A3	0.22	−0.11	−2.94**	−0.76	0.15*	0.41	−0.62	−0.85**	−0.38	−1.15**	0.15
C11 × A1	−0.52*	0.17	0.29	−0.54	0.14*	0.73	−1.15	0.20*	−0.22	−0.33	−1.26**
C11 × A2	0.47*	−0.18	−0.10	−0.26	0.09	−0.41	−0.83	−0.07	−0.50*	0.03	0.37
C11 × A3	0.05	0.02	−0.19	0.80	−0.23**	−0.33	1.97*	−0.13	0.72**	0.29	0.88
C12 × A1	0.46	−0.05	−0.01	0.67	−0.07	−1.54	−0.76	−0.05	−0.32	−0.02	−0.15
C12 × A2	−0.27	−0.44	−0.97	0.25	−0.09	0.25	0.35	0.12	0.19	−0.06	0.19
C12 × A3	−0.19	0.49	0.98	−0.91	0.15*	1.29	0.41	−0.07	0.13	0.07	−0.04
C13 × A1	−0.17	−0.43	−0.34	0.36	−0.04	−0.13	0.32	0.14	−0.16	−0.01	−0.20
C13 × A2	−0.16	0.28	−0.04	−0.17	0.20**	0.08	−0.32	0.08	0.69**	0.42	−0.31
C13 × A3	0.33	0.15	0.37	−0.19	−0.16**	0.06	0.00	−0.21	−0.52*	−0.41	0.52
C14 × A1	−0.07	0.06	0.26	0.19	−0.32**	−0.30	−0.40	−0.43**	0.53^*^	−0.10	1.89**
C14 × A2	−0.13	0.08	0.12	0.07	0.20**	0.88	0.39	0.37**	−0.20	0.62*	−0.81
C14 × A3	0.20	−0.14	−0.38	−0.26	0.12	−0.58	0.00	0.06	−0.32	−0.52	−1.09
LSD (0.05)	0.99	1.32	1.72	1.87	0.53	1.91	2.01	0.67	1.03	1.10	1.45

^a^LpH, low pH.

^b^Op, optimum pH.

^*^, ^**^ Significant at the .05 and .01 probability levels, respectively.

A combination of the highest number of significant positive and negative SCA effects under low pH was observed at Makoholi 1, closely followed by Makoholi 2 and Bindura 2. The highest number of a combination of significant positive and negative SCA effects under optimum soils was detected at Makoholi 2. The lowest number of a combination of significant positive and negative SCA effects were observed under low pH at Marondera and Domboshava and Bindura 1, and under optimum soils at Marondera and Bindura 1 and 2. No significant SCA effects were detected at Domboshava under optimum soils. The cross with the highest significant and positive SCA effect under low pH was C10 × A1 followed by C9 × A3, whereas under optimum soils it was C11 × A3 followed by C14 × A1. The crosses C9 × A3, C10 × A1, and C14 × A2 were associated with the highest number of significant and positive SCA effects under low pH and were therefore the best cross combinations under this environment.

## 4 DISCUSSION

Variation in yield performance of crosses within and across soil treatments at each site and across seasons and locations demonstrated the differential response of genotypes at different levels of soil pH. These results are consistent with findings by Dewi‐Hayati et al. (2014), Tandzi et al. (2015), and Gudu, Maina, Onkware, Ombakho, and Ligeyo (2001), who observed genotypic variation in maize grain yield under low soil pH and Al toxicity. Mean grain yield of the crosses significantly declined under low pH compared with optimum soils across environments and seasons per location, which indicates that soil acidity adversely affected maize grain yield. These findings were consistent with the results of similar studies of maize yield in acid soils (Ali et al., 2012; Borrero, Pandey, Ceballos, Magnavaca, & Bahia Filho, 1995; Ceballos, Pandey, Narro, & Pérez, 1998; Pérez Velásquez et al., 2008; Dewi‐Hyati et al., 2014). Low pH reduced grain yield of crosses by 11–37% at individual environments and 13–31% across environments and seasons. The current study clearly illustrates the sensitivity of maize grain yield to low pH stress. These findings are consistent with previous studies of maize yield in acid soils (Ali et al., 2012; Borrero et al., 1995; Ceballos et al., 1998; Pérez Velásquez et al., 2008).

The significant effects of environment and genotype for grain yield observed indicated that a combination of distinctive test environments and adequate genetic variability among the inbred lines was used. These results were in agreement with findings from other studies (Badu‐Apraku, Oyekunle, Akinwale, & Aderounmu, 2013; Badu‐Apraku, Oyekunle, Akinwale, & Lum, 2011), where they concluded that such a combination of factors would facilitate and enhance good progress for improvement in grain yield under abiotic stress and optimum conditions. Significant genotype and GCA variance for lines and testers of grain yield across environments and seasons revealed that there was scope for improvement of this trait through selection under low‐pH and optimum soils. These finding were consistent with the results obtained by Badu‐Apraku et al. (2013) in their study of the inheritance of grain yield and other traits under stress and nonstress environments. Significant differences among genotype and line GCA variance also implied that there were differences in the performance of the inbred lines in hybrid combinations, which agrees with findings by Makumbi, Betrán, Bänziger, and Ribaut (2011). A combination of significant GCA variance for lines and testers and SCA variance for grain yield suggested that both additive and nonadditive gene action were important in the inheritance of this trait under low‐pH and optimal soils. These findings were similar to the results obtained by Abrha, Zeleke, and Gissa (2013), Gowda, Kage, Lohithaswa, Shekara, and Shobha (2013), Narro et al. (2000), and Pérez Velásquez et al. (2008), where they concluded that both additive and nonadditive gene action were important in determining maize grain yield under acid and non‐acid soils. This information is extremely important in formulating effective breeding strategies whose objective is to develop inbred lines and hybrids with desirable adaptation to a wide range of soil pH levels.

The greater relative importance of additive gene action over non‐additive gene action observed in this study is in agreement with findings from other studies (Makumbi et al., 2011; Malvar et al., 2008; Welcker et al., 2005). These results, however, are contrary to findings of other authors (Badu‐Apraku et al., 2013; Badu‐Apraku, Lum, Akinwale, & Oyekunle, 2011; Guei & Wassom, 1992), who reported that there was a predominance of nonadditive gene action for maize in conditioning grain yield under abiotic stress.

Environment SS were eight times larger than genotype SS, which suggests that the environments used in this study were very diverse, and therefore large differences among environmental means were responsible for most of the variation in grain yield. Significant GCA × environment and SCA × environment interaction suggests that GCA effects associated with parents and SCA effects of their corresponding hybrids were not consistent over environments and seasons. This was similar to results obtained by Makumbi et al. (2011), whose conclusion was that using different parents at different locations for hybrid development and selection would be more effective and successful when based on performance across environments.

Line C8 from DR&SS had the highest significant positive GCA effects for grain yield under low pH. Therefore, the line would be suitable, together with its derivatives, as potential donor stocks and source populations for initiation of an acid‐soil tolerance breeding program by the institution. Tester A1 was the best general combiner under low pH, since it not only produced the highest positive GCA effects among testers but also had 44% of its total positive GCA effects observed under this environment. Furthermore, its superiority as a good general combiner was shown by its contribution of 69% of all positive GCA effects under both environments. Tester A2 was a good general combiner, since 33% of its significant GCA effects under low pH were positive, which validates its potential role as a donor for acid‐soil tolerance in maize breeding. Testers A1 and A2 originate from CIMMYT's acid‐soil tolerance breeding program in Colombia (L. Narro, personal communication, 2010). Line C12 was the best general combiner under low pH, whereby 80% of its total positive GCA effects, coupled with absence of negative GCA effects, were observed under this environment. It was closely followed by lines C6 and C14, where ∼50% of their respective total positive GCA effects were under low pH. Among lines, C14 and C6 were the best general combiners under low pH and optimum soils, since they contributed 20 and 17%, respectively, of all the positive GCA effects under both soil conditions. Line C10 and tester A3 were the poorest general combiners, since the former was associated with the highest number of negative GCA effects, whereas A3 gave 60% of all negative GCA effects under both soil conditions.

Inbred lines with favorable GCA effects for grain yield are likely to successfully contribute favorable alleles in a recurrent selection program and could be useful as parents in constituting synthetic populations suitable to be improved for abiotic stress tolerance (Makumbi et al., 2011). Close examination of pedigree information for lines C6 and C12, whose total positive GCA effects under low pH were 80 and 50%, respectively, indicate that they were extracted from the same population, 00SADVE, which might have alleles for acid‐soil tolerance. Tester A1 clearly proved that it was bred not just specifically for acid tolerance but equally for adaptation to optimum soils, since it was outstanding as the best overall general combiner under both environments. It is therefore suitable for development of new acid‐soil‐tolerant inbred lines with the yield potential to also excel as parents under optimum soils. Morello, Ferreira, and Filho (2002) identified six maize populations with desirable GCA effects for potential use in the constitution of new composites with good tolerance to soil acidity and low fertility. Testers A1 and A2 are ideal and effective as donors for use in the introgression of alleles for acid tolerance into local germplasm by virtue of their ability to give a high number of significant desirable GCA effects under low pH.

The crosses C10 × A1, C9 × A3, C14 × A2, C11 × A3, and C14 × A1 were associated with the most desirable SCA effects for grain yield. Significant positive SCA effects for grain yield were detected in 61% of the crosses, whereas 39% had significant negative SCA effects. This contradicts findings of Ali et al. (2012), where 48.3% of their crosses had positive SCA effects and 51.7% showed negative SCA effects. Ceyhan, Avci, and Karada (2008) detected significant positive SCA effects for grain yield in some of their crosses, which indicated the importance of nonadditive gene action for this trait. They therefore advised that more emphasis should be placed on specific cross combinations followed by selection in progenies instead of resorting to GCA through mass selection. In the current study, crosses with desirable SCA effects for grain yield were associated with high per se mean yields across treatments, locations, and seasons, which showed high correlation between positive SCA effects and high grain yield. This agrees with Surya and Garguli (2004), who also obtained a combination of desirable SCA effects and high per se yield performance.

All crosses (except C8 × A3) were composed of good × good and good × poor general combiners. This was consistent with the findings of Gowda et al. (2013), who suggested that involvement of at least one good general combiner was critical for obtaining good specific cross combinations. According to these authors, superiority of cross combinations from good × poor combiners could be attributed to interaction between positive alleles from good combiners and negative alleles from poor combiners as parents. The high‐yielding ability of such crosses is nonfixable and therefore can only be effectively exploited through heterosis breeding.

Significant positive SCA effects and high per se yield performance were obtained from 42% of the crosses under low pH across environments and seasons. Therefore, priority should be given to hybridization of specific crosses with desirable SCA effects, followed by yield selection per se in progenies as proposed by Ceyhan et al. (2008). However, where both additive and nonadditive gene action are important for the expression of maize grain yield.
